# Infographic: 2-year efficacy, durability and safety of intravitreal faricimab with treat-and-extend dosing up to 16 weeks in neovascular age-related macular degeneration (pooled results from TENAYA and LUCERNE)

**DOI:** 10.1038/s41433-024-03209-8

**Published:** 2024-07-10

**Authors:** Naomi Wijesingha, Aachal Kotecha, Philippe Margaron, Sobha Sivaprasad

**Affiliations:** 1 UCL Institute of Ophthalmology, London, UK; 2 Moorfields Eye Hospital NHS Foundation Trust, London, UK; 3 Roche Products Ltd., Welwyn Garden City, UK; 4 F. Hoffmann-La Roche Ltd., Basel, Switzerland

**Keywords:** Retinal diseases, Outcomes research

**Fig. 1:**
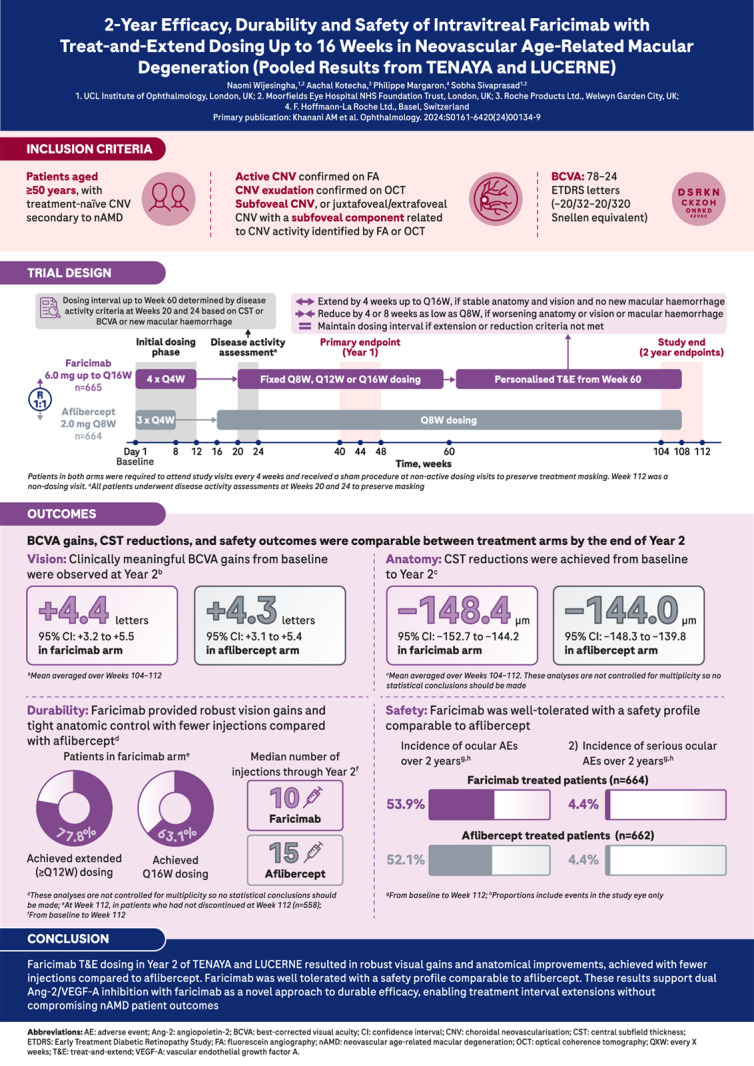
Pooled 2-year results from two Phase 3 trials (TENAYA and LUCERNE) of faricimab, a bispecific antibody targeting both Ang-2 and VEGF-A, in patients with nAMD. These results support dual Ang-2/VEGF-A inhibition with faricimab as a novel approach to durable efficacy, enabling treatment interval extensions without compromising nAMD patient outcomes.

