# Correction: Infographic: 2-year efficacy, durability and safety of intravitreal faricimab with treat-and-extend dosing up to 16 weeks in neovascular age-related macular degeneration (pooled results from TENAYA and LUCERNE)

**DOI:** 10.1038/s41433-024-03309-5

**Published:** 2024-08-26

**Authors:** Naomi Wijesingha, Aachal Kotecha, Philippe Margaron, Sobha Sivaprasad

**Affiliations:** 1 UCL Institute of Ophthalmology, London, UK; 2 Moorfields Eye Hospital NHS Foundation Trust, London, UK; 3 Roche Products Ltd., Welwyn Garden City, UK; 4 F. Hoffmann-La Roche Ltd., Basel, Switzerland

**Keywords:** Retinal diseases, Outcomes research

Correction to: *Eye* 10.1038/s41433-024-03209-8, published online 10 July 2024

The article “Infographic: 2-year efficacy, durability and safety of intravitreal faricimab with treat-and-extend dosing up to 16 weeks in neovascular age-related macular degeneration (pooled results from TENAYA and LUCERNE)”, written by Naomi Wijesingha, Aachal Kotecha, Philippe Margaron and Sobha Sivaprasad, was originally published electronically on the publisher’s internet portal on 10 July 2024 without open access. With the author(s)’ decision to opt for Open Choice the copyright of the article changed on 13 August 2024 to © The Author(s) 2024 and the article is forthwith distributed under a Creative Commons Attribution 4.0 International License, which permits use, sharing, adaptation, distribution and reproduction in any medium or format, as long as you give appropriate credit to the original author(s) and the source, provide a link to the Creative Commons licence, and indicate if changes were made.

The images or other third party material in this article are included in the article’s Creative Commons licence, unless indicated otherwise in a credit line to the material. If material is not included in the article’s Creative Commons licence and your intended use is not permitted by statutory regulation or exceeds the permitted use, you will need to obtain permission directly from the copyright holder.

To view a copy of this licence, visit http://creativecommons.org/ licenses/by/4.0/.

